# The three-legged stool of understanding metabolism: integrating metabolomics with biochemical genetics and computational modeling

**DOI:** 10.3934/microbiol.2018.2.289

**Published:** 2018-04-08

**Authors:** Diana M. Downs, Jannell V. Bazurto, Anuj Gupta, Luis L. Fonseca, Eberhard O. Voit

**Affiliations:** 1 Department of Microbiology, University of Georgia, Athens, GA, 30602, USA; 2 Department of Biological Sciences, University of Idaho, Moscow, ID, 83844, USA; 3 Department of Biomedical Engineering, Georgia Institute of Technology, 950 Atlantic Drive, Suite 2115, Atlanta, GA, 30332-2000, USA

**Keywords:** genomics, metabolic pathway, metabolomics, systems analysis

## Abstract

Traditional biochemical research has resulted in a good understanding of many aspects of metabolism. However, this reductionist approach is time consuming and requires substantial resources, thus raising the question whether modern metabolomics and genomics should take over and replace the targeted experiments of old. We proffer that such a replacement is neither feasible not desirable and propose instead the tight integration of modern, system-wide omics with traditional experimental bench science and dedicated computational approaches. This integration is an important prerequisite toward the optimal acquisition of knowledge regarding metabolism and physiology in health and disease. The commentary describes advantages and drawbacks of current approaches to assessing metabolism and highlights the challenges to be overcome as we strive to achieve a deeper level of metabolic understanding in the future.

## 1. Background

Historically, our understanding of metabolism was achieved by piecing together data meticulously obtained by reductionist biochemical and genetic approaches, predominately in bacteria. While unquestionably successful, these efforts required considerable research hours and vast resources. Further, they tended to advance the field incrementally over a span of decades. In the current era of rapid technological advances, some consider this pace unacceptably slow, and a cost-benefit analysis raises the question whether modern technologies, and in particular various omics methods, can accelerate the rate of progress in metabolic and physiological understanding without compromising the high-quality standards of traditional methods.

The disciplines of genomics and metabolomics continue to evolve and have proven to be game changers in biological data acquisition, but they are not without shortcomings. While these technologies can generate datasets of unprecedented volume and oftentimes technically excellent quality, the demonstration of biological relevance and reliability has not always been maintained. Genomic approaches come with the caveat that gene sequences are several steps removed from the actual function of metabolic and physiological processes, which implies uncertainty regarding genome-based metabolic insights. Indeed, targeted studies have suggested that transcriptomic and proteomic changes often do not correlate well [1]–[5]. Metabolomics, while providing data closer to cellular function, is currently limited by difficulties in reproducibly quantifying metabolite concentrations across replicates and experiments. Also, metabolomic data alone are not particularly informative regarding the processes governing a pathway system. We suggest that future understanding of metabolic network structure and function is best achieved by the simile of a three-legged stool: an approach that tightly combines classical biochemical-genetic experiments with global omics techniques and a pipeline of computational methods of analysis. The analogy appears to be suitable since in the absence of any one leg, the stool cannot stand, but when all legs are present, a three-legged stool is exceptionally stable. Similarly, the simile aptly describes the need for successful integration of the three indicated approaches in efforts to understand metabolic systems.

## 2. Metabolism is central to understanding biology

A well-balanced and robust network of biochemical processes is a fundamental feature of any living system. Metabolism, directly or indirectly, affects all cellular functions and is thereby at the very heart of our understanding of life. While genes often receive credit or blame in health and disease, perturbations in metabolism, resulting in the accumulation of a toxic metabolite, or the lack of a needed metabolite, are associated more directly with pathology [6],[7]. In fact, a fundamental question in biology is how the relatively small number of products encoded in the genome can generate the diverse and seemingly unlimited number of phenotypes observed in organisms from bacteria to humans. In other words: How are the products that are encoded in the genome (genotype) functionally integrated and regulated to result in metabolic pathways and processes that together generate the vast variety of robust and efficient physiologies (phenotypes) found in living cells?

The current body of metabolic knowledge is the culmination of decades of research hours, in which genetic and biochemical experiments, both *in vivo* and *in vitro*, defined biochemical reactions, their regulation, and associations with other cellular processes. This collective scientific effort generated advances that were then painstakingly pieced together into an enormous fundus of scientific articles and textbooks that describe our cumulative metabolic knowledge. More recently, these data have been morphed into comprehensive websites like KEGG [8], MetaCyc [9], BRENDA [10]. These printed and electronic resources are invaluable for studies that involve biological systems. The current data inventories are comprehensive but certainly not complete; a realization that emphasizes the continued need for the discovery of new metabolic components and their modes of regulation.

An important contribution of experimental approaches is the definition of paradigms, patterns that correlate with the presence of certain enzymes, gene regulation, or other cellular properties. Paradigms might include structural features of a protein that predict the involvement of a specific cofactor or catalytic activity, DNA binding sites that suggest inclusion in a regulon, or phenotypes that predict the presence of a pathway or function. Significantly, a paradigm has hallmark features that can be used to predict (or at least postulate) the presence of a regulon and/or metabolic pathway. For instance, if addition of exogenous cAMP reverses a nutritional phenotype, one immediately invokes the paradigm of catabolite repression [11]. Such a finding then predicts, and indirectly supports, a number of pathway properties that had not been directly tested. The value of a well-documented paradigm is that it minimizes (but does not eliminate) the experimental work necessary to arrive at reliable conclusions, thereby reducing the need to reinvent the wheel in studies of each new organism. While this strategy naturally comes with some caveats, and exceptions do occur, it has turned out to be an effective approach to advance our knowledge of diverse organisms.

## 3. Generation of fundamentally new knowledge remains challenging, and essential

Extrapolations from paradigms relieve some experimental redundancy. However, further accumulation of new knowledge is critical for defining additional paradigms that move our understanding of metabolism forward. This new knowledge is highly dependent on experimental rigor and data validation, but the required data acquisition is likely to be costly and time-consuming. The advent of omics technologies brings hope that transcriptomic and metabolomic data will offer new metabolic insights in a more cost- and resource-efficient manner, as long as it is ascertained that these data are of sufficient rigor and pass biological vetting, as described below.

The increasing ability to generate system-wide snapshots of the inner workings of a cell has raised our expectations of what is possible and led some bold scientists to conclude that a full understanding of the living cell is just around the corner. The truth might not be quite as rosy. On the positive side, vast datasets can be generated with comparatively little effort. They shed light on correlations between data points in a way that was not possible with the piecewise approach that dominated experimental biology prior to the advancement of global technologies. Computational methods of machine learning and big data analysis can quantify these often complicated, nonlinear correlations and allow the investigator to make predictions and generate hypotheses in the context of the whole cell. The value of such a systems-level approach is that it can uncover trends that would not be seen if one was looking at the read-out of one or a few metabolites or enzymes. No doubt, pursuing hypotheses that were extracted from global datasets has collectively resulted in the generation of a substantial body of new biological knowledge.

On the negative side, the vast increase in data generated per unit of effort sometimes leads to a relaxed standard for proof of biological significance, as if somehow the sheer mass of data would obviate the need for deeper biological queries that are more difficult and time consuming to pursue. This risk of a decreased demand for rigor can negatively affect our mechanistic understanding by overlooking salient data, or worse, drawing invalid conclusions that may take hold and become propagated in the literature. Moreover, the dissemination of data from high-throughput technologies has resulted in a subliminal trend where one is tempted to accept the results of large-scale experiments without appropriate critique or analyses of potential caveats. This ready acceptance raises a number of questions: Are we willing to accept conclusions based on cursory analysis, just because so many data points appear to support them? Due to the fact that many conclusions are now drawn by machine learning experts, is there a noticeable risk that the data are biologically inconsistent with our existing body of knowledge? Are we getting intellectually lazy?

If conclusions based on the global results of an omics analysis appear to conflict with our intuition or knowledge base, what should our response be? Are we skeptical of the new technology and experimental design, which may include presumably error-free robotic execution of the mechanics of the experiment? Or do we question past results obtained by traditional experimentation? If we choose the latter, does it represent an inappropriate infatuation with newness and technological advancement? Clearly, it is necessary to be aware of the pitfalls and caveats of all methods, but it seems that they are particularly insidious in the realm of omics. As a remedy, we suggest here a strategy of combining traditional and omics approaches in efforts to understand metabolism in the most efficient and reliable manner. Specifically, the significant potential of omics approaches should be tempered by rigorous, intrinsic quality checking and by extrinsic vetting against the traditional body of biological knowledge.

## 4. Defining metabolic potential from gene sequences: pros and cons

Improvements in sequencing, and correlations between sequence and function, allow the rapid annotation of genomes, which in turn can be used to predict the metabolic processes that occur in poorly characterized, or even non-culturable microorganisms. This inference strategy, which has become very popular, is based on the reasonable assumption that metabolic systems in related species are more similar than different, an assumption generally supported by genomic and functional analyses. Indeed, this strategy has resulted in efficient predictions regarding the metabolic capacity (phenotype) of an organism based on the enzymes encoded by its genome (genotype). For instance, based on our understanding of the TCA cycle and the enzymes required for its function, we can classify organisms as competent or incompetent to utilize succinate based on the genomic presence of the required enzymes. Moreover, this conclusion is made without an experiment, a pure culture or even a complete genome. Of course, these annotation-dependent, functional predictions and metabolic models are not without caveats. But even if they were 100% accurate, our understanding of the relationship between genotype and phenotype would remain critically incomplete, as we discuss next.

At issue are two core shortcomings of the genome-homology-based strategy of metabolic reconstruction and its ability to provide physiological insights. First, the existence of a particular gene, coding for an enzyme of interest, is a necessary but not sufficient condition for the enzyme to be active, and the litany of possible regulatory interventions, including induction, repression, post-translational modification, and others, in the chain of processes from DNA to active protein is long. Also, it is essentially impossible to detect single amino acid exchanges that could not only influence enzyme activity, but even substrate specificity. In addition, annotation and metabolic modelling are often strongly impaired in understudied bacterial and archaeal phyla due to high numbers of hypothetical proteins. As a consequence, the inference of metabolic network architecture from gene sequence, transcription profiles or proteomics is quite indirect. Second, assuming the isolated enzymes do have the function ascribed by their genomic annotation, using this activity to define metabolic potential incorrectly assumes that, when the parts are the same, the metabolic network structure and its function will be the same. It has by now become clear that this is not always a valid assumption, reducing the confidence with which metabolism can be reconstructed simply from genome information [12]–[14]. It seems fair to say that, at present, we do not have the means to predict metabolic state or function reliably from knowledge of the network components encoded in the genome.

So, what is lacking? The inference from a genome yields the prediction of a metabolic network. This network contains nodes (metabolites) and connecting edges (reactions; enzymes). The connectivity is of obvious importance, but it is insufficient to understand the true capacity of the system, because critical contributors to the metabolic system structure cannot yet be extracted from genome sequence. Critically, the amount and type of material that normally flows, or can potentially flow, through a particular enzymatic reaction is not discoverable from the genome sequence. Secondly, genome annotation depends on known pathways and paradigms and is therefore not able to predict new or recruited pathway structures. Finally, one might add that it is difficult to retrieve the regulatory structure of a pathway solely from genome sequence.

**Figure 1.**
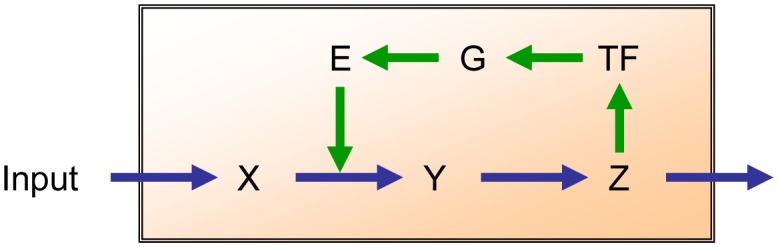
Illustration pathway with feedback. A linear metabolic pathway (blue arrows) generates metabolite Z, where Z exerts feedback (green arrows) onto the reaction step between metabolites X and Y. This feedback could also occur in the form of competitive or allosteric inhibition. Here, it is not purely metabolic, but affects the production of a transcription factor TF, which promotes expression of gene G, which codes for enzyme E that catalyzes the conversion of X into Y. How does the pathway respond to a change in input? Intriguingly, the answer is complicated: without numerical values determining flux rates and effector strengths, it is impossible to predict its responses. The metabolites may assume a new steady state, they may exhibit damped oscillations, or they may even assume a mode of ongoing (limit cycle) oscillations. Adapted from [15],[16].

Two specific examples will illustrate these challenges. First, a system that feeds back on itself (e.g., Figure 1) may exhibit qualitatively different responses, depending on the kinetic properties of the involved enzymes. With exactly the same structure, but with different values of the various kinetic parameters associated with enzymes and metabolite pool sizes, the system may respond to a change in input by: moving to a different state; briefly over- or undershooting; exhibiting damped oscillations; or entering a pattern of sustained oscillations [15],[16]. Because the distinguishing numerical details cannot be gleaned from genome information, the true phenotypic behavior of the pathway cannot be predicted.

**Figure 2.**
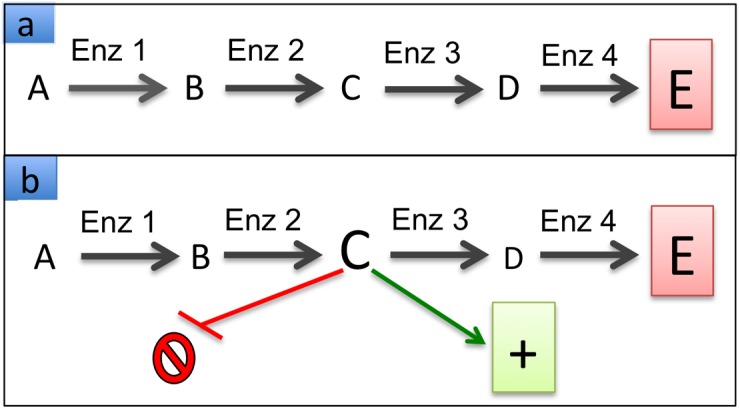
Schematic illustration of pathway interactions in two organisms. A generic pathway containing enzymes Enz1–Enz4, generating metabolites A–D and forming product E is shown. Panel a: diagram of the pathway in Organism 1 at equilibrium growth. Panel b: in Organism 2, metabolite C accumulates during equilibrium growth and affects the function of other pathways negatively or positively.

The second example illustrates that predictions of pathway integration, and thus network behavior, are not reliable if any of the relevant metabolites have roles outside the primary pathway. Consider the diagram in Figure 2, which represents an actual metabolic pathway, although with significant simplifications [17]–[21]. The figure schematically compares two similar organisms. The crucial point of the comparison is that phenotypes are governed by the integration and regulation of all metabolic components, not simply by the presence of the component enzymes.

Figure 2a depicts the flux through the pathway during balanced growth of Organism 1, where metabolites A–E are present at their nominal concentrations. Genotypic analysis would reveal that enzymes Enz1–Enz4 are indeed encoded by the genome and correctly predict that Organism 1 can synthesize product E. Figure 2b represents essentially the same pathway in Organism 2. Genome analysis would again demonstrate the encoding of enzymes Enz1–Enz4, and support the notion that Organism 2 can synthesize compound E. However, it is quite possible that the pathway flux during balanced growth is different from the flux in Figure 2a, and that, for instance, the concentration of metabolite C is significantly elevated. This quantitative difference could be due to any number of possibly subtle changes in the relevant enzymes (e.g., less active enzyme Enz3, more active enzyme Enz1 or Enz2, both effects simultaneously, or other constellations). Importantly, the altered flux pattern and elevated level of metabolite C can have profound phenotypic implications by inhibiting processes or systems outside the pathway or activating others.

Thus, although the genomes of the organisms represented in Figure 2a, b encode exactly the same enzymes, standard genomic analyses would incorrectly predict the two organisms to have the same phenotype: Yes, they can both synthesize product E, but the analysis would fail to recognize any phenotypic consequences of the altered level of metabolite C, which could be significant for the fitness of the organism.

The two examples illustrate the importance of an additional layer of information flow that, on top of the stoichiometric network of connections, determines metabolic behavior. The subtle intricacies of this layer of control and regulation are responsible for the plasticity and adaptability that is characteristic of metabolic systems and critical for their responsiveness to perturbations due to external or internal signals [15]–[17],[22]–[27]. Critically, these subtleties are not detectable in the network structure of a pathway, i.e., its connectivity, but are fine-tuned, quantitative features of the mechanisms that control them. While intuition fails in this situation, higher-order metabolic properties are, at least in theory, deducible by combining information from gene expression with global measures of metabolites and the support of efficacious computational models.

## 5. Current metabolomics: pros and cons

Broadly speaking, two complementary strategies are being pursued in metabolomics today. The first seeks to detect as many compounds in a biological sample as possible, despite the fact that many, if not most, cannot be identified as known metabolites. Nevertheless, this strategy can provide evidence of metabolic divergence, for instance by comparing the metabolomes of a healthy cell and a cancer cell [28]–[30]. The alternative to this global approach involves the more modest goal of monitoring and quantifying fewer compounds—maybe at the order of 100—of known identity [31],[32]. Clearly, this alternative robs metabolomics of some of its appeal, but it is beneficial for interpretation and further analysis of questions such as those posed herein. Together, the two metabolomics approaches have the potential to facilitate understanding of the properties of metabolism. After all, the metabolic content of the cell at any given time reflects the consequence of all upstream activities, i.e., transcription, translation, flux control, pathway integration, as well as much of the downstream activity that is manifest in a healthy or abnormal phenotype.

At least in principle, metabolomics can determine whether a specific metabolite is present, and at what concentration. Alas, what we need to understand metabolism is not only the concentration of each metabolite, but also details of the enzyme kinetics of each step. Whereas metabolomics appears to allow for the former need to be achievable in the future, the latter goal is currently impossible on a global scale, largely because each enzyme has distinct features and catalyzes different reactions. The hope is that the global snapshots of metabolites provided by metabolomics, combined with targeted traditional experiments, will one day provide the data needed to develop computational models of metabolism, and provide new insights into enzyme kinetics and pathway dynamics.

## 6. The need for computational analysis

In the past, paucity of data was usually the bottleneck of metabolic modeling; clearly, this situation has changed [33],[34]. The sheer amount of information in a typical transcriptomics or metabolomics dataset leaves little doubt that computational means are needed to capture these datasets, organize them, and make them accessible. Effective mathematical and computational models are required to present the data in a useful format and allow them to be understood and queried. Analysis based on these models allows us to identify inaccuracies and misconceptions in our current understanding of metabolic pathways, discover rules that govern the integration of high- and low-flux metabolic pathways, and predict the global effects of pathway disruption or metabolite accumulation.

The first computational requirement for addressing a dataset is statistical analysis. The omics revolution has mandated expansions of biostatistics in entirely new directions. One deals with the fact that, in contrast to traditional experiments that investigated one or a few features with many replicates, metabolomic and genomic experiments target hundreds or thousands of features, often with comparably few replicates [35]. The second direction of statistical analysis evolved in response to exploratory omics experiments where it is a priori unclear what one might discover in the data output. The computational methods for extracting information from noise in these datasets fall into the domains of statistical machine learning, artificial intelligence, and big data analysis [36]. More often than not, these patterns are not identifiable with the unaided human mind as they consist of complex, typically non-linear correlations among the data. If a newly discovered pattern can be interpreted in terms of the biology of the investigated phenomenon, it ideally leads to a new hypothesis that correlates datasets to each other in a mechanistic, explanatory manner [37].

Hypotheses regarding the mechanisms governing a phenomenon may be straightforward, complicated, or possibly even counterintuitive. In the latter two cases, a casual mental analysis, or even a lab experiment, may not be sufficient to assess the veracity of the hypothesis. A potentially effective alternative of addressing this situation is the creation of an explanatory mathematical model, perhaps a dynamic model that is formulated as a set of nonlinear differential equations.

Because multiple models may be formulated for the same system, arguably the most important inputs into the model design process are available data and the specific questions to be answered by the model, which will mandate structural features in the model that constrain the range of possibilities to some degree. If we follow the pipeline from raw data via statistics and machine learning toward testable hypotheses, both data and questions are given; however, data and questions can just as well come from traditional experiments.

In addition to these considerations, a model structure must be chosen and implemented. The model structure includes variables that represent metabolites, enzymes, and modulators, as well as possibly other factors, and the equations that capture which of the variables contribute to changes in any of the variables over time. Typically, each equation contains terms contributing to increases (production) and decreases (utilization) of a variable. The existence or absence of these terms in each equation is dictated by the connectivity of the metabolic system. By contrast, the actual mathematical format of each term is a matter of debate, and while some defaults are being used time and again, it is in truth almost always impossible to choose objectively from among a variety of formulations [38]–[40]. The final input to the model design consists of parameter values, such as reaction rates and inhibition constants, which convert the model from a symbolic structure into a model specifically addressing the given pathway system and possibly a given dataset. The actual process of determining suitable parameter values that are predictive for given datasets is often complicated [41]–[43].

Once a dynamic model has been diagnosed and fine-tuned, it is ready for analysis, which raises the question: what are reasonable expectations from a metabolic model? A few typical expectations are the following: the model should permit an adequate account of various structural and regulatory features and be capable of faithfully capturing the movement of metabolites throughout the pathway system. It should fit the data reasonably well, and explain responses of the system to perturbations. Finally, the model should be able to make reliable qualitative or quantitative predictions regarding untested situations, explain why organisms with the same genetic or metabolic components can still act differently, and be useable to compute a state of the pathway system that is optimal with respect to a given objective.

Of course, today's models often fall short of these idealized features. These shortcomings are partially due to overly limited, incomplete, and noisy data. Additionally, we often do not realize the importance of factors outside, or even inside, the pathway that have an effect on pathway components. On the mathematical side, it is not clear what functions best represent metabolic processes. Mass-action, Michaelis-Menten, and power-law models are often used as defaults, but there is no objective guarantee that any of these are generally optimal or correct; in fact, many other representations have been proposed [38]. For related reasons, most models should be expected to fail when asked to predict responses to drastic changes in the organism or its environment. Such failure is often due to unknown and/or ill-characterized response systems or internal constraints and the fact that all mathematical representations are approximations which cannot be extrapolated arbitrarily with satisfactory accuracy. Furthermore, methods of parameter estimation have greatly improved in recent times, but they are still far from ideal or even failsafe for large systems at the scale of omics data.

In spite of these unsurprising limitations, metabolic modeling has become a valuable complement to experimentation and will continue to improve the characterization of regulated pathway systems of increasing size. One should also mention that a quickly expanding repertoire of metabolic modeling methods permits the use of different types of data, including metabolic responses to gene knock-downs [44], mass spectrometry results, metabolic time series obtained from NMR measurements [45],[46], in addition to more traditional results.

Some of the models of the recent past have been confirmatory, by integrating available information into a model that fitted data reasonably well, and was explanatory or predictive. In particular, many models have demonstrated that important metabolic components were missing or misrepresented, which led to necessary experimental investigation and adjusted explanations. In other cases, unknown modes of regulation were predicted by the model analysis, and specific single and double knockdowns were proposed, purely based on computational results, to achieve desirable phenotypes.

In a different vein, the field of flux balance analysis, with hundreds of publications, has been targeting the optimization of metabolic yields or fluxes, typically based on static, whole-organism models derived by inference from genome information [47],[48]. To a lesser degree, but with the promise of increased accuracy and reliability, fully regulated, dynamic models have been used for similar optimization purposes [49],[50]. In both cases, the models predicted optimal system responses to targeted alterations in enzyme amounts or activities.

## 7. Bacterial case study

Bacterial systems provide several advantages over other organisms for increasing our understanding of the relationship between genotype and phenotype. In particular, the ability to define the genotype of the organism quickly, coupled with ease of phenotypic analysis, provides a rich system to obtain corollary data and test hypotheses rapidly. Significantly, these studies have broad applicability, since the resulting paradigms will be relevant to other organisms due to the conservation of key aspects of central metabolism across the domains of life.

The purine/histidine/thiamine (PHT) node of metabolism in *Salmonella enterica* was recently described as a model system in a proof-of-principle study to integrate quantitative metabolite measurements and biochemical-genetic experimental approaches with mathematical modeling [51]. The lessons learned from this study can be summarized by two points.

First, generating a symbolic model of the PHT node without parameter values was straightforward. However, when critical data were collected from the literature to facilitate the design of an initial, fully parameterized computational model, lack of information regarding key enzymes and the diversity and unequal quality of the data available in the literature stalled these efforts. We turned to metabolomics data with the assumption that they would provide a single source of high-quality metabolite concentrations with which to refine the model. This assumption was not borne out, and initial work to query the metabolomes of relevant strains under appropriate conditions highlighted weaknesses in this approach [51].

Secondly, while several positive correlations between metabolomics data and past knowledge were extracted from these datasets, they relied on general, qualitative trends across metabolomics data from several biological replicates and experiments. Further, the differences we found were seldom supported by a measure of statistical quality, so that it was difficult to assign significance to metabolic differences that, according to past work, were assumed to be true. Ultimately, the limitations in the experimental results were found to be due to significant, unexplained variation among replicates [51]. The conclusion was that current metabolomics techniques appeared to be nominally valuable in confirming biological conclusions, but less reliable in identifying unexpected changes in metabolic state. It is highly likely that, as the technology improves and biological variation can be minimized or somehow taken into account, it will become feasible to use these approaches to generate data of the quality and quantity needed for mathematical models, but we have not yet reached this point.

## 8. Future

At present, it is difficult to obtain reliable, quantitative metabolomics data across samples and experiments using LCMS platforms. It is worth noting that transcriptomics as a technology went through growing pains, and standards and statistical criteria eventually arose in the field as the technology gained traction. Since those early days, the quality and reproducibility of the data have increased and made transcriptomics a critical and reliable tool in biomedical research. There is every reason to believe the same will be true for metabolomics as the field grows and experimental and computational biologists continue to refine laboratory approaches and statistical analyses.

A challenge with current LCMS metabolomics methods is the necessary disruption of the intact cell which generates unknowable effects on the metabolites in total. The future development of non-invasive *in vivo* methods will be critical to quantify cellular metabolism accurately. Such approaches will eventually replace the best current efforts to replicate the pertinent features of the intracellular milieu, which we do not fully understand.

In this vein, *in vivo* Nuclear Magnetic Resonance (NMR) spectroscopy provides an exciting possibility for non-invasive assessments of metabolite concentrations (e.g., [52]–[56]). Although these techniques are currently limited by the amounts of metabolites and numbers of species present, its applicability is certain to improve. Indeed, the combination of *in vivo* NMR approaches with traditional experimental approaches and sophisticated computational modeling has the potential to bring new insights into metabolism, as some case studies demonstrate [45],[46],[53],[57],[58].

In summary, to understand the relationship between genotype and phenotype we must account for the connectivity between the metabolic components encoded in the genome as well as the cellular context that influences these connections and gives rise to phenotypic plasticity. Given the steep trajectory of technical innovation in the recent past, it is likely that new approaches will support future efforts to understand the subtleties of metabolism. Beyond technological innovations, it is mandatory that a workforce is created and nurtured that is well-versed in both experimental and computational work, trained to bridge the two fields, and able to communicate results effectively. Traditional biological science education programs are often devoid of a computational component, but a change in today's curricular standards will pay dividends as we strive to understand the complex system of metabolism that is central to all living cells.

## 9. Final thoughts

A vision on the distant horizon, considered the Holy Grail in biology, could take the form of an animation of the inner workings of the cell, based on a comprehensive understanding of the complex systems of metabolism and physiology, and illuminating the processes in the Central Dogma as they proceed in pseudo-3-dimensional space. While we are far from realizing this vision, continuing efforts toward a deeper understanding of metabolism will require multiple, complementary approaches to advance the field toward this goal. The growing number of available data will demand not only methods of statistical data analysis and machine learning, but also the conversion of simple correlations among data into mechanistic, computational models that offer explanations and suggest novel hypotheses that are testable with combined experimental and mathematical methods.
